# Functional interaction between Vangl2 and N-cadherin regulates planar cell polarization of the developing neural tube and cochlear sensory epithelium

**DOI:** 10.1038/s41598-023-30213-x

**Published:** 2023-03-08

**Authors:** Tadahiro Nagaoka, Tatsuya Katsuno, Kyoka Fujimura, Kunihiro Tsuchida, Masashi Kishi

**Affiliations:** 1 Division for Therapies Against Intractable Diseases, Center for Medical Science, Fujita Health University, 1-98 Dengakugakubo, Kutsukake-Cho, Toyoake, Aichi 470-1192 Japan; 2 Center for Anatomical, Pathological and Forensic Medical Researches, Graduate School of Medicine, Kyoto University, Yoshida-Konoe-Cho, Sakyo-Ku, Kyoto, 606-8507 Japan; 3 Neuroscience Laboratory, Research Institute, Nozaki Tokushukai Hospital, 2-10-50 Tanigawa, Daito, Osaka 574-0074 Japan

**Keywords:** Cell biology, Developmental biology

## Abstract

Although the core constituents of the Wnt/planar cell polarity (PCP) signaling have been extensively studied, their downstream molecules and protein–protein interactions have not yet been fully elucidated. Here, we show genetic and molecular evidence that the PCP factor, Vangl2, functionally interacts with the cell–cell adhesion molecule, N-cadherin (also known as Cdh2), for typical PCP-dependent neural development. Vangl2 and N-cadherin physically interact in the neural plates undergoing convergent extension. Unlike monogenic heterozygotes, digenic heterozygous mice with *Vangl2* and *Cdh2* mutants exhibited defects in neural tube closure and cochlear hair cell orientation. Despite this genetic interaction, neuroepithelial cells derived from the digenic heterozygotes did not show additive changes from the monogenic heterozygotes of *Vangl2* in the RhoA–ROCK–Mypt1 and c-Jun N-terminal kinase (JNK)–Jun pathways of Wnt/PCP signaling. Thus, cooperation between Vangl2 and N-cadherin is at least partly via direct molecular interaction; it is essential for the planar polarized development of neural tissues but not significantly associated with RhoA or JNK pathways.

## Introduction

Planar cell polarity (PCP) refers to the coordinated alignment of cellular polarity across multidimensional tissues. The molecular mechanism of planar polarization is essentially conserved across the animal kingdom^1^. In vertebrates, PCP governs tissue morphogenesis, including neurulation of the embryonic neural plates^2,3^, formation of the auditory sensory epithelia of the inner ear cochleae^4,5^, and branching morphogenesis of epithelial tissues, such as the lung and kidney tissues^6,7^. The core components of PCP signaling, including Frizzled, Dishevelled, Prickle, and Vang-like (Vangl) proteins, are required for any of the planar polarization, and these molecules reside at the boundaries between neighboring cells with asymmetric protein localization. Indeed, a crucial function of PCP signaling is the regulation of the cell–cell adhesion in polarizing tissues.

One of the cell–cell adhesion molecules regulated by the PCP signal is cadherin, which is linked to actin filaments via catenin sub-membranous proteins. Cytoskeletal regulation of the cadherin molecules is therefore controlled by the Rho–Rho kinase pathway of PCP signaling; PCP signal-regulated Rho kinase phosphorylates the myosin-binding subunit of protein phosphatase 1 regulatory subunit 12A (PPP1R12A, also known as Mypt1) and induces actomyosin contraction, thereby determining the localization of cadherins^8^. When cell–cell junctions are under stress, Rho GTPases also mediate the endocytic regulation of classic cadherins^9^. However, it is unknown how the universal regulator of endocytosis, the Rab family small GTPase, is involved in PCP-related internalization of classic cadherins. Further, the mechanism by which cadherin molecules detached from catenin/actin cytoskeleton are regulated remains unclear.

We previously reported the postsynaptic localization of Vang-like 2 (Vangl2)^10^ planar cell polarity protein at mammalian excitatory synapses^11^, where it regulates the distribution of the synaptic cell–cell adhesion molecule, cadherin 2 (CDH2, also known as N-cadherin), via Rab5-dependent endocytosis. This internalization is required for normal synaptogenesis and is elicited by the direct molecular interaction of Vangl2 with N-cadherin, which is detached from β-catenin. However, whether this molecular function of Vangl2 is essential for typical PCP-dependent neural development processes, such as neurulation and cochlear epithelial alignment, remains unclear, and the relevance of the direct regulation of N-cadherin by Vangl2 in non-canonical Wnt signaling also warrants further investigation. In the present study, we analyzed the genetic and molecular interactions between Vangl2 and N-cadherin during neural development to confirm the crosstalk between the two. Furthermore, we investigated whether the RhoA–Mypt1 actomyosin or c-Jun N-terminal kinase (JNK)–Jun nuclear pathway of Wnt/PCP signaling was involved in their functional interaction. Our results suggest that the direct regulation of N-cadherin by Vangl2 constitutes the Wnt/PCP signaling pathway that plays a key role in mammalian neural development.

## Results

### Physical interaction between Vangl2 and N-cadherin in the neural tube

**Figure 1:**
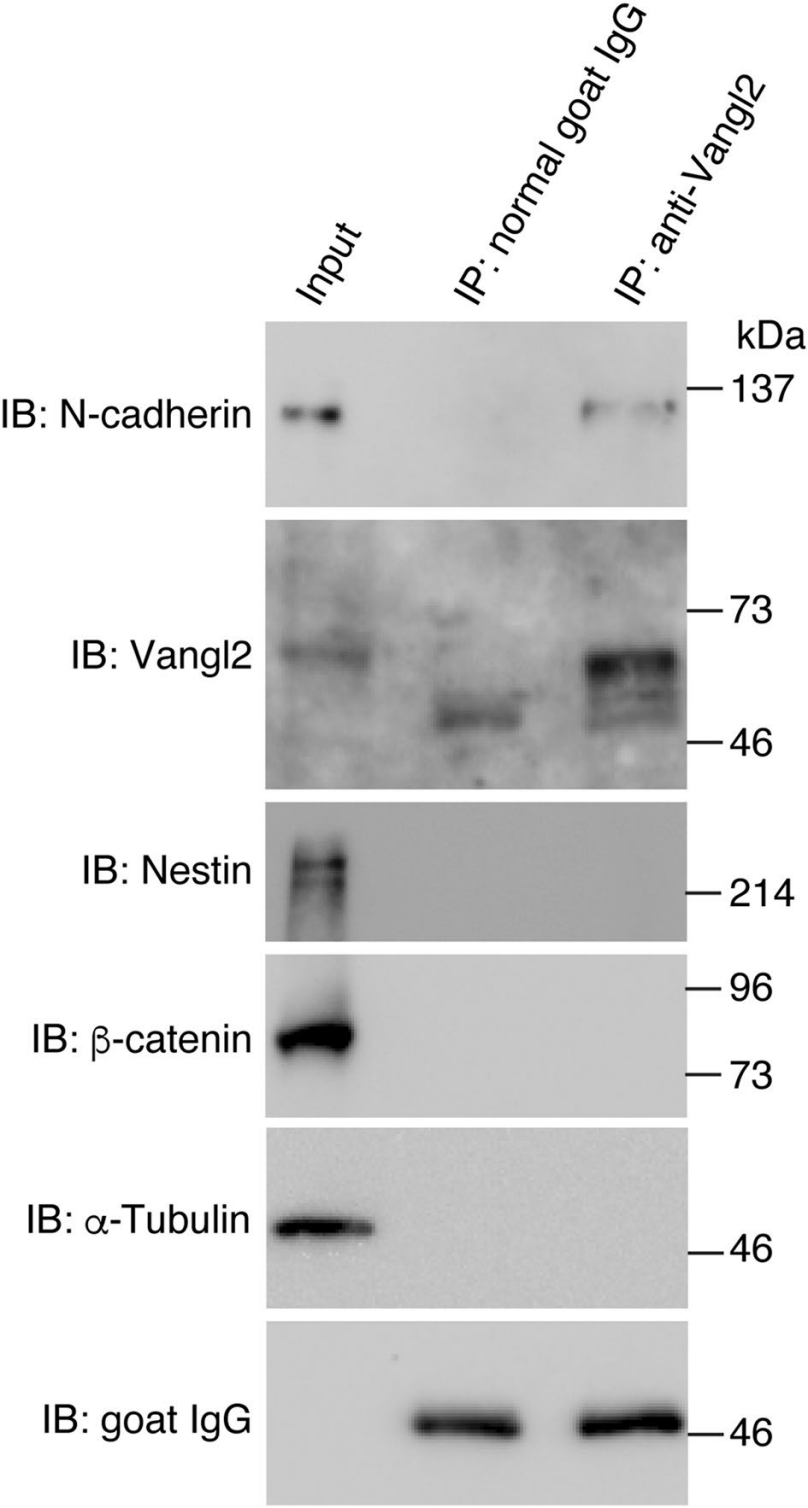
N-cadherin—Vangl2 association in neural tube cells. Immunoprecipitation using normal goat IgG or goat anti-Vangl2 antibodies from neural tube cell lysates derived from E9.5 embryos. Western blot analysis of the input (1.7%) and IP (~ 50%) using the indicated antibodies confirmed the specific co-immunoprecipitation of N-cadherin by anti-Vangl2 antibodies.

**Figure 2:**
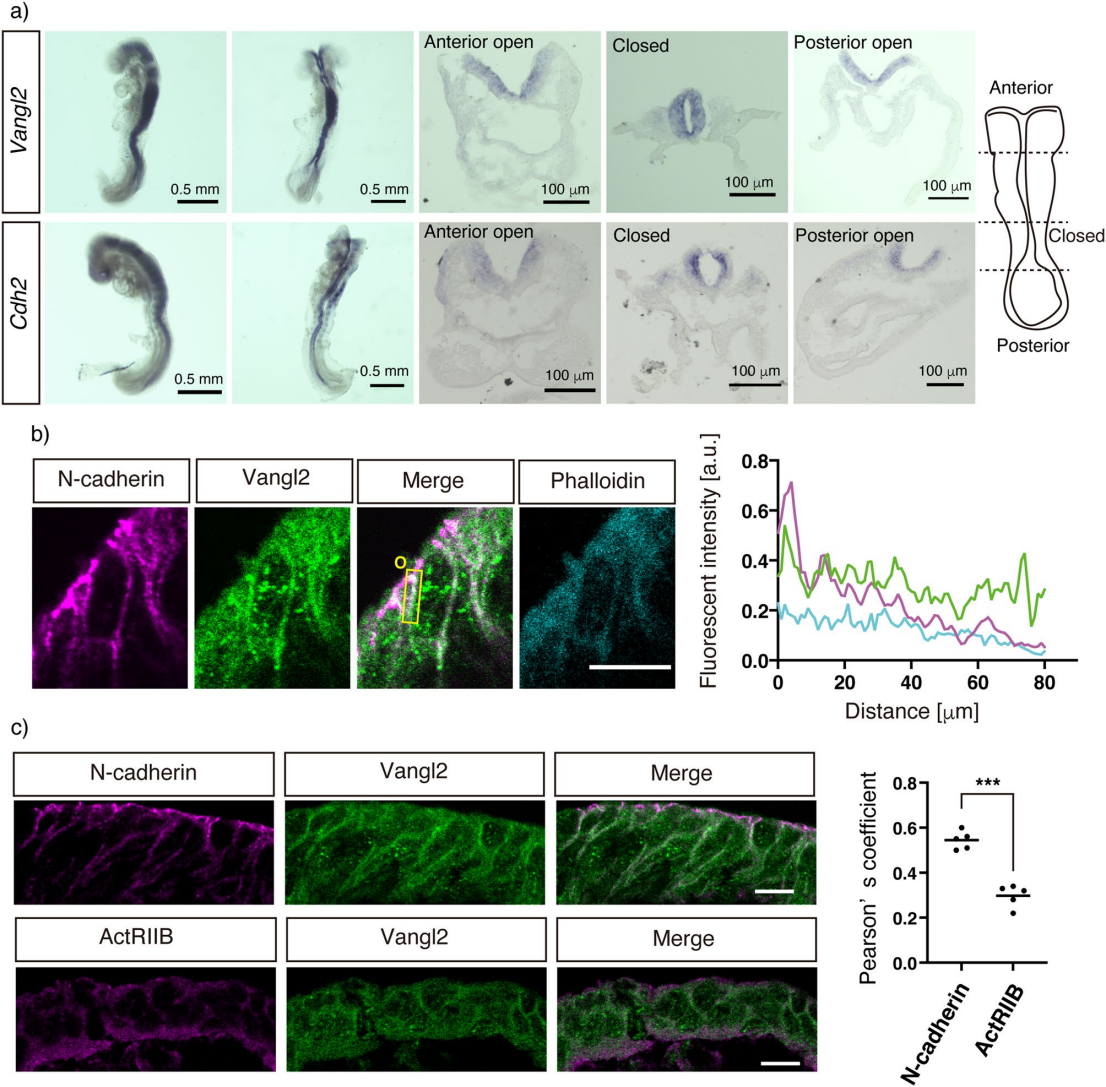
Vangl2 and N-cadherin expression in mouse embryos. (**a**) Whole mount in situ hybridization with antisense probes for *Vangl2* and *Cdh2* in mouse embryos at E8.5 (lateral view, left panel; dorsal view, second left panel). The transverse images of the embryos sectioned at the indicated levels with the dashed lines (cartoon on the right) are also presented (middle panels; the anterior neural plate, second right panels; closed neural tube, right panels; posterior neural plate). *Vangl2* and *Cdh2* are predominantly expressed in the neural plate and neural tube. (**b**) Coronal section of the mouse neural plate using anti-N-cadherin (magenta), anti-Vangl2 (green), and phalloidin (cyan). The plot profile of each fluorescent signal intensity in the depicted rectangle (yellow box) is shown on the right with the respective color. The line graphs indicate the partial co-localization of Vangl2 and N-cadherin. O; origin. (**c**) Double immunofluorescence analysis of Vangl2 and N-cadherin and that of Vangl2 and activin receptor type IIB (ActRIIB). The Pearson’s coefficients of their co-localization measured using the software Coloc 2 are presented as dot graphs. The coefficient of Vangl2/N-cadherin is significantly higher than that of Vangl2/ActRIIB. Significant differences between groups were calculated using the Student’s *t*-test (*** *P* < 0.0001). Scale bars: 0.5 mm (whole mount) and 100 μm (sectioned) in a, 10 μm in b and c.

We previously established the importance of physical interactions between Vangl2 and N-cadherin in the regulation of dendritic spine formation in excitatory synapses^11^. To further investigate the role of this interaction in the formation of other tissues via PCP-dependent processes, we investigated the physical interaction between Vangl2 and N-cadherin in the neural tube of mouse embryos. Neural tube closure typically occurs from E7.5 to E10 in mice^12^, and a co-immunoprecipitation (co-IP) assay performed in primary culture cell lysates of neural tubes isolated from E9.5 mouse embryos revealed the direct interaction of N-cadherin with Vangl2, but not with β-catenin, the scaffold protein of classical cadherins (Fig. 1). The cell lysate used in the co-IP assay was validated via immunoblotting with an anti-Nestin antibody, a marker of neural progenitor cells, including neural tube cells^13^. Furthermore, *Vangl2* and *Cdh2* were found to be predominantly expressed in neural folds and spinal cord cells of E8.5 embryos via whole-mount in situ hybridization (Fig. 2a). An additional analysis of sectioned embryos using the same technique revealed almost homogenous expression of these genes from the cranial to caudal regions. Immunofluorescent staining further revealed the co-expression of Vangl2 and N-cadherin proteins in the rostral and caudal neural folds as well as the closed spinal cord cells of E8.5 embryos (Fig. 2b). Vangl2 and N-cadherin were also found to partially co-localize in the neural fold and spinal cord cells (Fig. 2c; Supplementary Fig. 1a–c). The Pearson’s coefficient of Vangl2 and N-cadherin co-localization was 0.53 ± 0.05, which was significantly higher than that of Vangl2 and the activin receptor type IIB, which is expressed in neural tube cell membranes (0.32 ± 0.05, p < 0.0001) (Fig. 2c)^14^. The anti-Vangl2 signal was blocked by incubation with the Vangl2 antigen peptide used to raise the antibodies but not with the anti-Vangl1 antigen peptide. This result indicated the specific recognition of Vangl2 proteins by the anti-Vangl2 antibodies (Supplementary Fig. 1d). These results indicate that Vangl2 and N-cadherin co-localize and physically interact in the neural plates undergoing PCP signal-dependent convergence and extension for the development of neural tubes.

### Genetic interaction between Vangl2 and Cdh2 during neural tube closure

**Figure 3:**
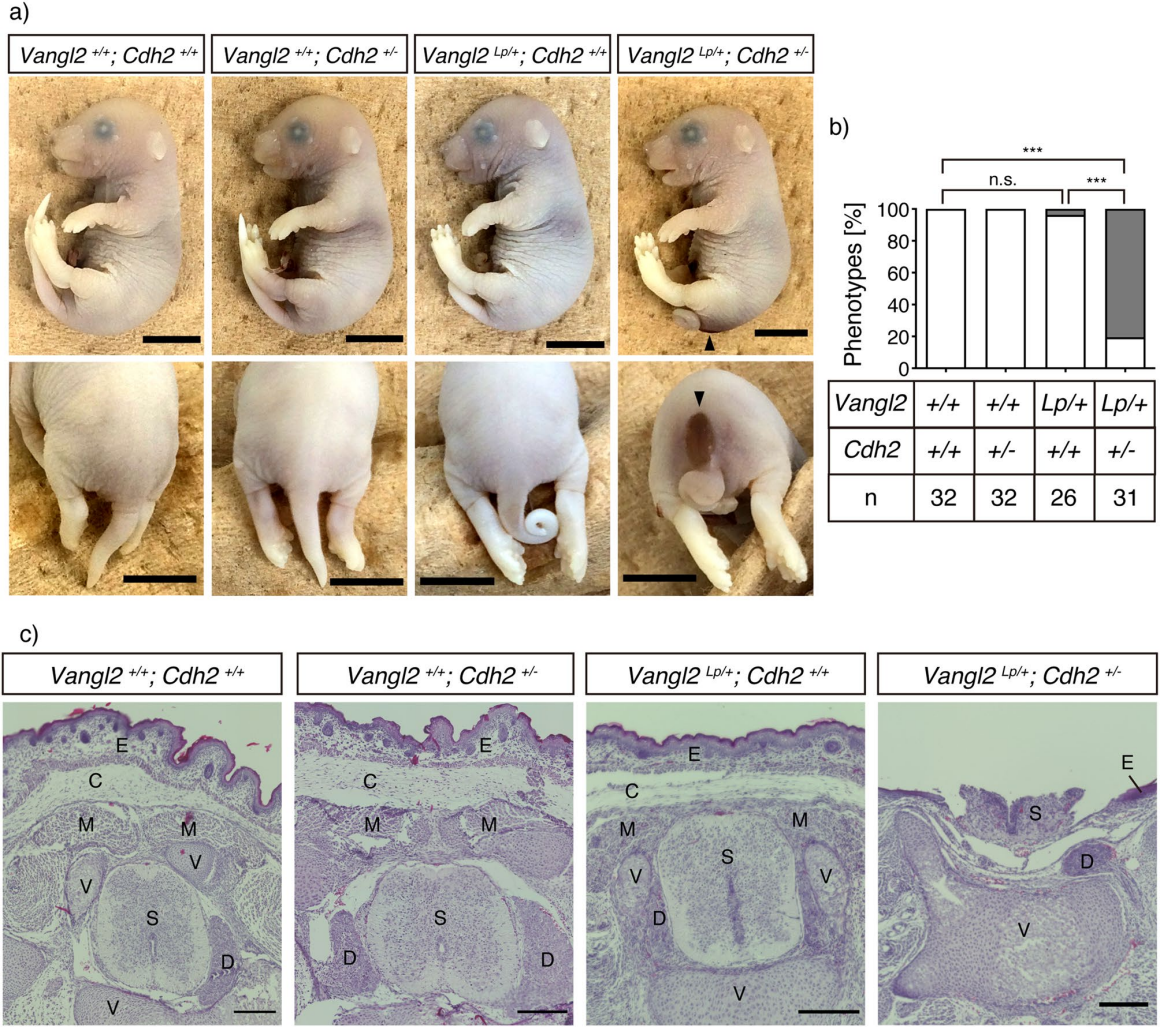
Genetic interaction between *Vangl2* and *Cdh2* in neural tube closure. (**a**) E18.5 embryos with the indicated genotypes are presented. The *Vangl2 *^*Lp/*+^*; Cdh2* ^+*/-*^ mice (the rightmost panels), exhibited an open neural tube phenotype similar to the human myelomeningocele. The arrowheads indicate the neural tube defects (NTDs). (**b**) Bar graphs showing the ratio of the embryos born with (grey) or without (white) the NTDs. The number of mice counted for each genotype is indicated below the bar graph. (**c**) Hematoxylin–eosin staining of the caudal portion of coronally sectioned E18.5 embryos. *E* epidermis, *C* connective tissue, *M* muscle, *V* vertebra, *S* spinal cord, *D* dorsal root ganglia. Scale bars: 0.5 mm in a, 200 μm in c. Significant differences between groups were calculated using Fisher’s exact test (*** *P* < 0.001). n.s.; not significant.

Given that *Lp* homozygotes (*Vangl2 *^*Lp/Lp*^) and *Cdh2* knockout (KO) homozygotes (*Cdh2 *^*-/-*^) are embryonically lethal^15,16^, *Vangl2*^*Lp/*+^*; Cdh2*^+*/*+^ mice (*Looptail* heterozygotes) were interbred with *Vangl2* ^+*/*+^*; Cdh2* ^+*/−*^ mice (*Cdh2* KO heterozygotes) to examine the genetic interactions between *Vangl2* and *Cdh2* in their embryos at E18.5. Intriguingly, *Vangl2*^*Lp/*+^*; Cdh2*^+*/−*^ (double heterozygous mutant) embryos displayed a spina bifida-like neural tube defects (NTDs) in the caudal region, and the frequency of its occurrence in the double heterozygous mutant embryos was significantly higher (80.6%, n = 31) than that in *Vangl2*^+*/*+^*; Cdh2*^+*/*+^ (wild-type, 0%, n = 32), *Cdh2* heterozygous KO mutant (0%, n = 32), and *Lp* heterozygous mutant (3.8%, n = 26) embryos (Fig. 3a,b). Furthermore, while hematoxylin–eosin staining confirmed the presence of an open caudal spine outside the slit-skin, the cephalad spinal cord was normally closed in double heterozygous mutant embryos (Fig. 3c). The sacral location of the open spine was further confirmed by micro-CT (Supplementary Fig. 2). The finding that spina bifida was frequently observed in digenic heterozygotes, but not in monogenic heterozygotes, indicated that the two molecules cooperate to ensure the neural tube closure, which is a typical PCP signal-dependent morphogenesis of vertebrate neural tissues.

### Genetic interactions between Vangl2 and Cdh2 do not affect the RhoA and JNK signaling pathways

**Figure 4:**
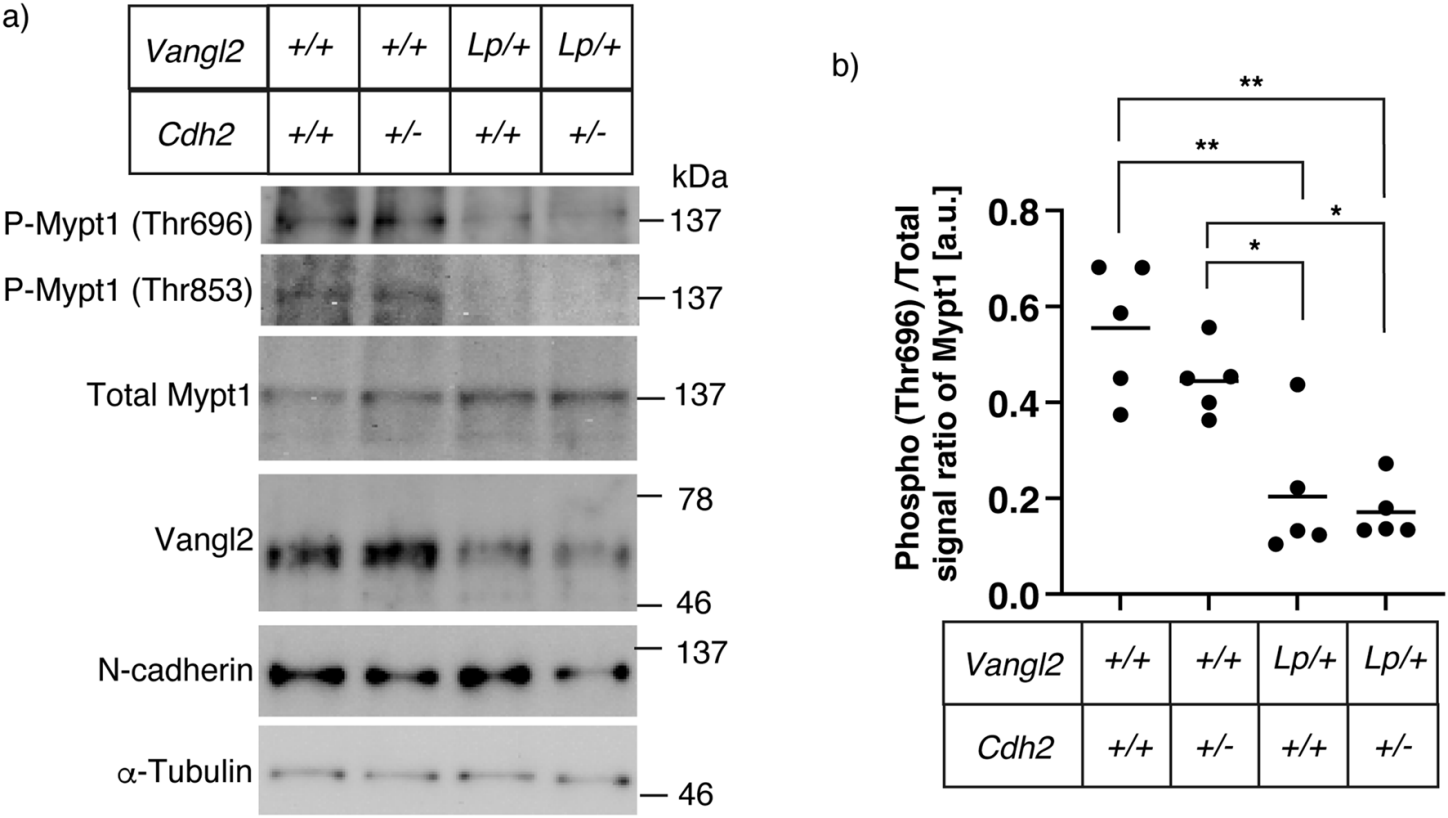
RhoA/ROCK signaling activity in the neural tube. (**a**) Western blot analysis of the neural tube cell lysates derived from E9.5 embryos with the indicated genotypes. Total and phospho-epitopes of Mypt1, the downstream of RhoA/ROCK pathway, were analyzed. (**b**) Point graph showing Thr696 phosphorylation levels of Mypt1. The ratios were calculated according to the signal strength of anti-phosphorylated Mypt1 and that of anti-total Mypt1. The phospho-levels were not significantly different between *Vangl2*^Lp/+^*; Cdh2*^+*/*+^ and *Vangl2*^Lp/+^*; Cdh2*^+*/−*^ embryos, although the levels for both embryos were lower than those of the wild type or *Vangl2*^+*/*+^*; Cdh2*^+*/−*^ embryos. The solid line indicates the mean of each genotype (n = 5). Significant differences between groups were calculated using Tukey’s multiple comparison test (*P < 0.05 or **P < 0.01).

Since PCP signaling activates the downstream molecules, RhoA^17^, Vangl2 may functionally interacts with N-cadherin via RhoA pathway. Mypt1, which regulates convergent extension by modulating actomyosin contractility acts downstream of RhoA in the process of neural tube closure^18,19^. The phosphorylation of Mypt1 (Thr696) in primary neural tube cell lysates as assessed by western blotting analysis in E9.5 embryos was significantly lower in the double heterozygous mutants (0.173 ± 0.060 a. u.) than in the wild-type (0.555 ± 0.138 a. u.; p = 0.0005) and *Cdh2* heterozygous KO mutants (0.445 ± 0.073 a. u.; p = 0.017) (Fig. 4a, b). However, no significant difference in Mypt1 phosphorylation levels between the double heterozygous mutant and *Lp* heterozygous embryos (0.204 ± 0.138 a. u.; p = 0.977) was evident. Because the pMypt1/Mypt1 ratio seemed to have reached its minimum in the Vangl2 heterozygotes, we could not reason that the NTDs caused by the additional *Cdh2* heterozygosity were because of further reduction in the phosphorylation levels of Mypt1. A similar result was observed in the western blot analysis using phospho-Mypt1 (Thr853) Abs; the phosphorylated Mypt1 protein in the cell lysates of Vangl2 heterozygotes was already undetectable. In these experiments, although *Cdh2* heterozygotes exhibited slightly reduced levels of Mypt1 phosphorylation, the difference was not significant (Thr696; 0.445 ± 0.073 a. u.; p = 0.403. Thr853; 0.507 ± 0.076 a. u.; p = 0.825).

**Figure 5:**
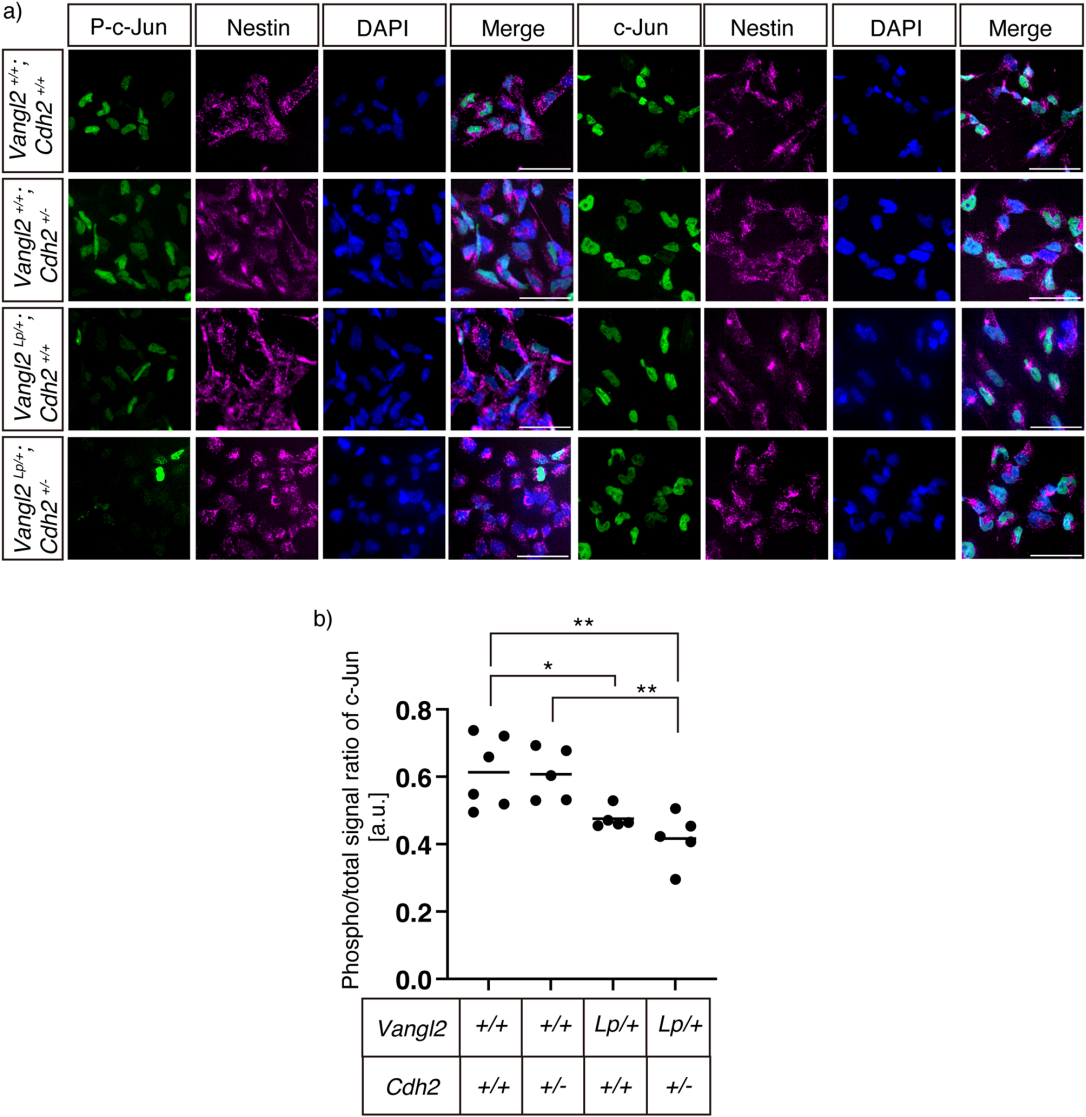
JNK signaling activity in neuroepithelial cells. (**a**) Immunofluorescence analysis of the primary neural tube cells derived from E9.5 embryos with the indicated genotypes. Cells were immunostained with either a combination of anti-phospho c-jun (green) and anti-Nestin (neural cells; magenta) or anti-c-jun (green) and anti-Nestin (magenta). Nuclei were stained with DAPI (blue). (**b**) Point graph showing ratio of immunofluorescent signals of phosphorylated c-Jun to those of the total in the Nestin-positive neuroepithelial cells. The phospho-levels were not significantly different between *Vangl2*^Lp/+^*; Cdh2*^+*/*+^ and *Vangl2*^Lp/+^*; Cdh2*^+*/−*^ cells, although the levels for both embryos were lower than those of the wild type or *Vangl2*^+*/*+^*; Cdh2*^+*/−*^ cells. The solid line indicates the mean (n = 5). Significant differences between groups were calculated using Tukey’s multiple comparison test (* *P* < 0.05 or ** *P* < 0.01). Scale bars: 50 μm.

We further evaluated the activity of JNK signaling, a nuclear pathway downstream of the Wnt/PCP pathway^20^ in cultured primary neural tube cells from E9.5 mouse embryos of each genotype. Cultures were fixed and immunostained with the anti-phospho-c-Jun or anti-c-Jun antibodies (Fig. 5a). The ratio of phospho-c-Jun signal intensity to total c-Jun signal intensity was calculated in cells confirmed to be of neural tube origin by nestin staining. The intensity of the phospho-c-Jun signal in neural tube cells derived from double heterozygous mutant embryos (0.417 ± 0.078 a. u.) was statistically similar to that of the *Lp* heterozygote embryos (0.476 ± 0.030 a. u.; p = 0.658), but significantly lower than that of the wild-type (0.613 ± 0. 106 a. u.; p = 0.0041) or *Cdh2* KO heterozygote embryos (0.607 ± 0.077 a. u.; p = 0.0075) (Fig. 5b).

Together, these results indicate that in the background of *Vangl2*^*Lp/*+^, the occurrence of spina bifida due to the heterozygosity of *Cdh2* was not evoked by changes in the signal strength of the RhoA-Mypt1 or JNK-Jun pathways. It is more likely that the reduced direct interaction between Vangl2 and N-cadherin, where the two examined pathways do not intervene, caused this NTDs.

### Genetic interaction between Vangl2 and Cdh2 in the orientation of inner ear hair cells

**Figure 6:**
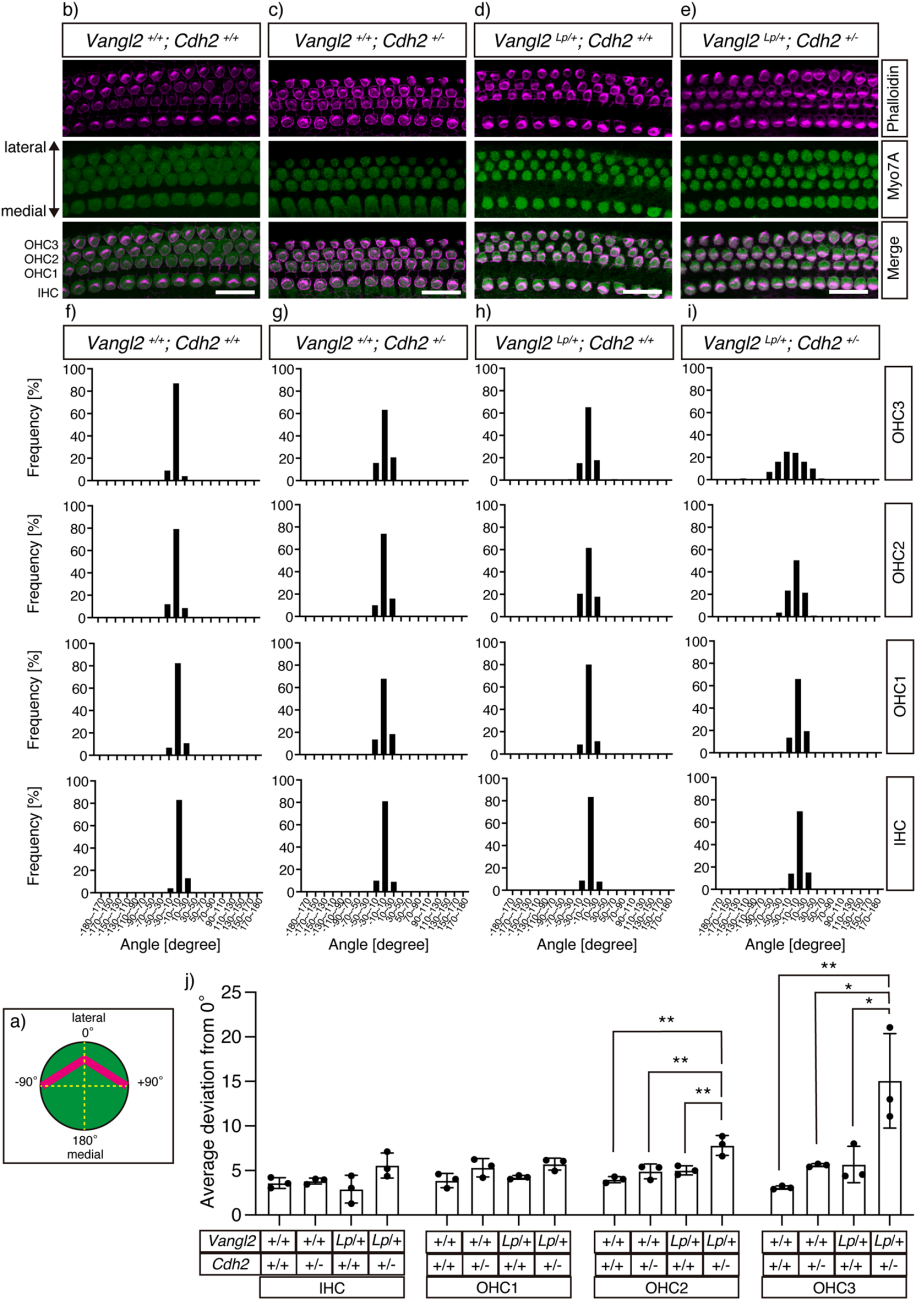
Genetic interaction between Cdh2 and Vangl2 in inner ear. (**a**) A diagram showing an example of a hair cell, whose bundle orientation is 0°. The depicted angles (-90°, 0°, + 90°, + 180°) were used as the standards to analyze the orientation of the stereociliary bundle of each hair cell. (**b–e**) Fluorescence images of the sensory epithelia in the E18.5 inner ear with the indicated genotypes. The stereociliary bundles were stained with phalloidin (magenta) and cochlear hair cells with antibodies against the hair cell marker Myo7A (green). (**f–i**) Bar graphs showing the frequency of stereocilia oriented toward given angle ranges. The patterns of angle distributions of four types of hair cells consisting of the sensory epithelia with the four genotypes are presented: (**f**) wild type, (**g**) *Vangl2* ^+*/*+^*; Cdh2* ^+*/-*^, (**h**) *Vangl2 *^*Lp/*+^*; Cdh2* ^+*/*+^ , and (**i**) *Vangl2 *^*Lp/*+^*; Cdh2* ^+*/-*^. (**j**) Bar graphs showing the average deviation of the angles of the stereocilia measured from 0°. The results of four types of hair cells derived from the four genotypes are presented. Note that the digenic mutants exhibit cumulative increase of angle deviation of the bundle orientation in OHC2 and OHC3. For each bar graph, more than 100 hair cells were subjected to the analysis (different litters, n = 3). Significant differences between groups that were calculated using Tukey’s multiple comparison test are marked with * (p < 0.05) or ** (p < 0.01). Scale bars: 20 μm.

The orientation of the sensory epithelium is regulated by PCP^1^. Therefore, we examined whether the cooperation between Vangl2 and N-cadherin was required for unidirectionally oriented development of the cochlear epithelium by measuring the angles of stereocilia in the inner ear hair cells (Fig. 6).

In E18.5 wild-type mice, we observed that approximately 80% of stereocilia were oriented within the ± 10° range toward the neural-to-abneural axis in all outer hair cell (OHC) rows (OHC1: 82%, OHC2: 79%, and OHC3: 87%) and inner hair cell (IHC) rows (83%) (Fig. 6b, f). We also found that 100% of the stereocilia were oriented within ± 30° of the axis in all row hair cells.

As previously reported for other mutations^21–23^, hair cells in the lateral side grow more sensitive to aberrant Vangl2 signaling. In the OHC3 row of *Cdh2* or *Vangl2* single mutants, the frequency of the stereocilia oriented within the ± 10° range was slightly reduced (*Cdh2* heterozygous KO:64%, *Lp* heterozygous mutant: 65%) and that of the ± 10–30° range was increased (*Cdh2* heterozygous KO:37%, *Lp* heterozygous mutant: 33%) (Fig. 6c,d,g,h). In the digenic mutants, the frequency of stereocilia within the ± 10° range was further reduced (24%), and that of the ± 10–30° range increased (41%). Moreover, we observed those within the ± 30–50° and ± 50–70° ranges in the digenic mutants (35 and 26%, respectively) (Fig. 6e,i). This genetic interaction was also observed in OHC2 row. With the single or double mutations, the frequency of stereocilia within the ± 10° range was reduced (WT: 79%, *Cdh2*: 74%, *Lp*: 62%, *Cdh2/Lp*: 50%), those within the ± 10–30° range increased (WT: 21%, *Cdh2*: 26%, *Lp*: 39%, *Cdh2/Lp*: 45%), and those within the ± 30–70° range appeared (WT: 0%, *Cdh2*: 0%, *Lp*:0%, *Cdh2/Lp*: 5%).

In OHC1 and IHC row, however, we could not observe significant genetic interaction in the frequency of the stereocilia oriented toward specific angle ranges (Fig. 6a–i). Calculation of the average deviation of stereocilia angles from 0° (Fig. 6j) also indicated insignificant differences among the IHC (wild type: 3.6 ± 0.6, *Cdh2* heterozygous KO: 3.8 ± 0.3, Lp heterozygous mutant: 2.9 ± 1.5, and double hetero mutant: 5.6 ± 1.4) and OHC1 (WT: 3.9 ± 0.8, *Cdh2* heterozygous KO: 5.3 ± 1.0, *Lp* heterozygous mutant: 4.2 ± 0.2, and double hetero mutant: 5.7 ± 0.7) of the four genotypes. In this evaluation, the average deviation in OHC2 (double hetero mutant: 7.8 ± 1.1 vs wild type: 4.0 ± 0.3, p = 0.001, vs *Cdh2* heterozygous KO:4.9 ± 0.8, p = 0.007, and vs *Lp* heterozygous mutant: 5.0 ± 0.5, p = 0.009) and in OHC3 (double hetero mutant: 15.0 ± 5.3 vs WT: 3.1 ± 0.2, p = 0.004, vs *Cdh2* heterozygous KO:5.6 ± 0.2, p = 0.015, and vs *Lp* heterozygous mutant: 5.7 ± 2.0, p = 0.016) of the double heterozygous mutant was significantly higher than that of the other three genotypes.

These results indicate that, in addition to neural tube closure, Vangl2 and N-cadherin cooperatively function for the proper orientation of their inner ear hair cells, at least in OHC2 and OHC3 row.

## Discussion

Digenic mutations in *Vangl2* and PCP-related genes frequently cause caudal NTDs in mice. For instance, 94% of digenic mice heterozygous for *Lp* and *Ptk7* exhibited this phenotype^21^. Similarly, with a heterozygous *Lp* background, 68% of heterozygous *Sec24B*, 17% of heterozygous *Syndecan-4* (*Sdc4*), and 55% of *Sdc4* homozygous mutants have the birth defects^22,23^. In the present study, 80.6% of the digenic mutant mice carrying *Lp* and *Cdh2* genes had NTDs. Based on these statistics, we propose that the cooperation of N-cadherin with Vangl2 is functionally significant, at least to a degree comparable to that of Sec24B or Sdc4 with Vangl2. As reported for *CELSR1*, *PTK7*, *DVL3*, and *SCRIB* gene mutations^24^, digenic or polygenic patterns of inheritance contribute to NTDs in humans. Exploration of *CDH2* mutations is warranted to decipher their role in NTDs, although there are no reports of their coexistence with PCP gene mutations in humans to date.

N-cadherin is expressed in the invaginating neural plate^25^ and plays a central role^26^ in neurulation. Our results revealed the expression levels of Vangl2 and N-cadherin along the neural plate and tube of E8.5 embryos (Fig. 2a). Furthermore, co-localization and specific interactions between Vangl2 and N-cadherin proteins (Fig. 2b) were demonstrated via immunofluorescence staining and co-IP from the lysate of neural tubes at E9.5 (Fig. 3). A previous study on neuroepithelium harboring mosaic deletions of *Vangl2* demonstrated the formation of N-cadherin-positive adherens junctions between *Vangl2* deleted cells and neighboring cells^27^. Since Vangl2 competes with β-catenin, a scaffold protein of classical cadherins, for N-cadherin binding^11^, the above-mentioned findings may be explained by the stabilization of the N-cadherin/β-catenin complex via Vangl2 deletion. In these mosaic neuroepithelia, the deletion of *Vangl2* genes might also reduce the internalization of N-cadherin and therefore increase its cell surface amount^11^. This leads to the stabilization of both the cis interaction of N-cadherin within the mutant cells (cell-autonomous) and the trans interaction between the adjacent cells (non-cell-autonomous). A similar mechanism may also underlie the PCP-dependent development of other neural tissues, such as the cochlear epithelium^28,29^, axonal growth cones^30^, and neural synapses^11^. Mosaic deletion of *Vangl2* genes in developing (growth cone) or developed (neural synapses) neural circuits would reveal whether the non-cell-autonomous features of PCP are conserved during the development of neural networks.

The RhoA-Rho kinase (ROCK) signaling pathway regulates convergent extension and initiation of neurulation^17^. We found that individual depletion of *Vangl2* affected RhoA-ROCK signaling; however, additional depletion of *Cdh2* did not result in a cumulative change in this pathway (Fig. 4). Likewise, the JNK–Jun signaling pathway regulates convergent extension^31^, and signal strength of the JNK signaling was not appreciably changed between the *Vangl2* heterozygous cells and those of *Vangl2*/*Cdh2* digenic heterozygotes (Fig. 5). Although there is a considerable difference between the two genotypes in the ratio of newborns with spina bifida, this difference is not associated with a change in RhoA–ROCK or JNK–Jun activity. Therefore, we reasoned that the Vangl2–N-cadherin pathway is not substantially linked with RhoA–ROCK or JNK–Jun signaling.

Shroom3, a downstream molecule of Dvl2-mediated PCP signaling^32^ that controls subcellular localization of ROCK^33^, has been reported to be a key regulator of neural tube closure^34^. Because the Dvl2–Shroom3 pathway determine ROCK function^33^, convergent extension movement of the neural plate should be controlled in parallel by the Dvl2–Shroom3 and Vangl2–N-cadherin pathways. In contrast, Shroom3 is known to regulate N-cadherin^34^. Therefore, we speculate that the Dvl2/Shroom3 complex controls N-cadherin localized on the Dvl/Frizzled side^1^, and Vangl2 controls it on the Vang/Prickle side of planar polarized neuroepithelial cells. N-cadherin localized on opposite sides might function downstream of distinct pathways. Reduction in N-cadherin levels on the Vang/Prickle side but not that on the Dvl/Frizzled side would functionally cooperate with the heterozygosity of *Vangl2* to develop NTDs. Further comprehensive analyses of the relationships between N-cadherin and other PCP-related molecules, including Shroom3, be it cooperative, competitive, or independent, will help unravel the detailed molecular mechanisms of neural tube closure.

The orientation of auditory sensory hair cells is disrupted in the OHC and IHC layers in *Vangl2 *^*Lp/Lp*^ homozygous cochleae^35^. Additionally, *Vangl2* knockout^36^ or conditional knockout lines^37^ demonstrate dominant disruption of the OHC3 orientation. The auditory sensory epithelia of *Lp* and *PTK7* double heterozygotes^21^ as well as that of *Lp* and *Sdc-4* mutants^23^ also show OHC3 misorientation. The orphan G protein-coupled receptor GPR156 and guanine nucleotide-binding proteins of the inhibitory alpha class (Gαi) axis regulate hair cell orientation under the transcriptional control of Emx2^38^. As GPR156 localization is altered in the hair cell junction of *Lp* mouse cochleae, it is possible that Emx2-GPR156-Gαi regulates hair cell orientation along the axis regulated by core PCP components. Although no adamant physical or functional correlation between N-cadherin and these molecules has been reported, for a more comprehensive understanding of the role of N-cadherin in neural tube closure, future investigations on whether N-cadherin is related to or independent of these molecules are required.

As for molecular expression, N-cadherin protein was abundantly found in the IHC layer and medial epithelial cells but scarce in the OHC1-3 layers (Supplementary Fig. 3), and our results were consistent with those of previous reports^28,39^. The low-level expression of N-cadherin protein in the OHC row might explain why disorganized orientation of the stereocilia was observed only in a similar area of the digenic mutant cochlea; the heterozygosity of *Cdh2* would easily make the N-cadherin levels insufficient for normal planar polarization in the OHC layers. In contrast, in the IHC layer, N-cadherin levels may still be sufficient for polarization in the digenic mutant because the original levels are very high. In future studies, further depletion of N-cadherin protein in the OHC layer might reveal the quantitative requirement of N-cadherin levels for the polarization of cochlear epithelia. The reason why OHC2 and OHC3 were the only rows with an appreciable phenotype remains unclear. Future comprehensive studies regarding the expression patterns of Wnts and their related molecules in the inner ear may explain the gradual strengthening of the phenotype from OHC1 to OHC3.

In conclusion, our findings revealed that functional interactions between *Vangl2* and *Cdh2* are involved in neural tube closure and auditory sensory epithelial orientation in mice. Further analyses of *CDH2* and its related molecules in patients may reveal the role of the PCP/N-cadherin pathway in the correct formation of neural tubes or cochlear epithelia in humans.

## Methods

### Animals

*Loop-tail* mutants of the *LPT/Le* stock (Stock No. 000220)^40^ and N-cadherin KO mutants of the *Cdh2*^*tm1Hyn*^ stock (Stock No. 003179)^16^ were obtained from Jackson Laboratory and backcrossed to C57BL/6 J mice for at least six generations. To obtain mutant mouse embryos, 8 weeks or older *Vangl2 *^*Lp/*+^ male and *Cdh2*^+*/−*^ female mice were interbred, and genotyping of embryos was performed using the yolk sac. *Loop-tail* genotyping was performed by amplifying the *Crp* microsatellite DNA marker as previously described^41^. N-cadherin KO genotyping was performed using previously described primers^16^. ICR mice were purchased from Japan SLC (Hamamatsu, Japan). All animal experiments were approved by the Institutional Animal Use and Care Committee of Fujita Health University (permission numbers AP17008 and APU19094) and were performed in accordance with the National Institutes of Health Guidelines for Care and Use of Laboratory Animals. All methods are reported in accordance with ARRIVE guidelines (https://arriveguidelines.org) for the reporting of animal experiments.

### Haematoxylin–Eosin staining

The lower bodies of E18.5 mouse embryos were fixed with 4% paraformaldehyde (PFA) at 4 °C overnight. Fixed samples were further processed using a Tissue-Tek VIP-VI automatic infiltration processor (Sakura Finetek, Tokyo, Japan) with a standard 19 h program, followed by transfer into moulds. The samples were subsequently embedded with paraffin wax and sectioned perpendicularly against the head–tail axis using a microtome (RM2125RT, Leica, Wetzlar, Germany) set at a thickness of 4 μm. Sectioned tissues were mounted on New Silane II coated glass slides (Muto Pure Chemicals, Tokyo, Japan) and incubated on a paraffin extension plate (Leica) at 45 °C overnight. Post deparaffinization, slides were soaked in Mayer’s haematoxylin solution for 10 min followed by washing with water for 20 min. Slides were then incubated in 1% Eosin Y solution for 5 min before visualization under an all-in-one microscope BZ-9000 (Keyence, Osaka, Japan).

### In situ hybridization

The target RNA probe sequences of *Cdh2*^42^ and *Vangl2*^43^ were amplified by PCR using KOD plus (Toyobo, Osaka, Japan), adenine tailed, and inserted into the pGEM-T easy vector (Promega, Madison, WI). DNA templates were amplified from pGEM-T-*Cdh2* or *Vangl2* by PCR, using M13 primers and ExTaq (TaKaRa Bio, Kusatsu, Japan). Amplified samples were purified using the QIAquick PCR Purification Kit (QIAGEN, Hilden, Germany). RNA transcription was carried out using the DIG RNA labelling mix (Roche) and T7 or SP6 RNA polymerase (Roche) at 37 °C for 2 h. The products were purified using NucleoSEQ columns (Macherey–Nagel, Düren, Germany) and an equal volume of formamide was added before storage at − 20 °C.

Mouse E8.5 Embryos were fixed in 4% PFA in phosphate buffered saline (PBS) at 4 °C overnight. The embryos were dehydrated using a methanol gradient (25, 50, 75 and 100% methanol) and 1% Tween-20 in PBS (PBT) for a period of 5 min in each solution. After rehydrating samples in a 75, 50 and 25% methanol/PBT gradient, samples were washed with PBT twice. Embryos were bleached with 6% hydrogen peroxide in PBT for 1 h at room temperature followed by washing with PBT thrice. They were subsequently incubated with 20 μg/ml proteinase K in PBT for 6 min at room temperature, followed by post-fixation treatment with PBT containing 4% PFA and 0.2% glutaraldehyde for 20 min and washing with PBT twice. Embryos were next washed with a 1:1 mixture of hybridization solution (50% formamide, 1% SDS, 50 μg/ml yeast tRNA, 50 μg/ml heparin, 5 × SSC, pH 4.5)/PBT and hybridization buffer for 10 min each at room temperature. The embryos were further incubated at 70 °C in hybridization solution for 1 h, followed by replacement of the solution with fresh hybridization solution containing the RNA probe and incubated overnight at 70 °C. The next day embryos were washed with 5 × SSC, pH 4.5 containing 50% formamide and 1% SDS thrice for 30 min each at 70 °C followed by three washes with 2 × SSC, pH 4.5, containing 50% formamide for 30 min each at 65 °C and two washes with RNase buffer containing 0.5 M NaCl, 1% Tween-20 and 0.1 M Tris–Cl, pH 7.5 for 5 min. Subsequently, embryos were incubated with 20 μg/ml RNase A for 30 min at 37 °C and washed thrice with Tris buffered saline containing 1% Tween-20 (TBST) at room temperature. The buffer was then replaced with TBST containing 10% sheep serum and 1% blocking reagent (Roche) for 1 h at room temperature, followed by incubation with anti-digoxigenin-AP Fab fragments (Roche) diluted in a blocking solution overnight at 4 °C. Embryos were then washed thrice with TBST for 5 min at room temperature, five times for 1 h at room temperature, and once overnight at 4 °C. The next day, embryos were washed with 100 mM Tris–Cl, pH 9.5 containing 100 mM NaCl, 1% Tween-20 and 2 mM Levamisole thrice for 5 min at room temperature, followed by replacement of the buffer containing 250 μg/ml NBT and 125 μg/ml BCIP. Whole-mount samples were visualized using a stereomicroscope (SZ9, Olympus, Tokyo, Japan) and images were recorded using a digital camera (DP-50, Olympus). Frozen sections were prepared by embedding stained samples in OCT compound (Sakura Finetek) and immediately freezing on frosted dry ice, followed by perpendicular sectioning against the anterior–posterior axis using a cryostat (CM1850, Leica) set at 10 μm thickness at − 20 °C. The sectioned samples were visualized under an all-in-one microscope BZ-9000 (Keyence, Osaka, Japan).

### Immunofluorescence

Mouse E8.5 embryos were embedded in OCT compound (Sakura Finetek) and frozen on finely crushed, dry ice before perpendicular sectioning against the anterior–posterior axis using a cryostat (CM1850, Leica) set at a thickness of 5 μm. Sectioned samples were adsorbed onto MAS glass slides (Matsunami, Kishiwada, Japan) and fixed in ice-cold methanol before they were blocked with PBS containing 10% FBS for 1 h at room temperature. Subsequently, sections were incubated with primary antibodies diluted in blocking buffer overnight prior to washing thrice with PBS and incubation with secondary antibodies conjugated to Alexa Fluor for 1 h. Samples were subsequently washed thrice with PBS and enclosed with FluorSave (Millipore, Burlington, MA, USA) before visualization under a confocal microscope (LSM710, Zeiss, Oberkochen, Germany). Co-localization of Vangl2 and N-cadherin proteins was evaluated by calculating the Pearson’s coefficient using the Coloc 2 plugin in Fiji (https://imagej.net/Fiji).

### Immunoprecipitation assay

Neural tubes were collected from ICR mouse E9.5 embryos and lysed in PBS containing 1% NP-40 and 10 μg/ml aprotinin. Immunoprecipitation and western blotting were performed as previously described^44^.

### Phospho-Mypt1 measurement

Neural tubes were collected from E9.5 embryos and lysed in PBS containing 1% NP-40, PhosSTOP (Roche, Mannheim, Germany), and 10 μg/ml aprotinin prior to analysis by western blotting. Chemiluminescent signal intensity was measured using the Fiji gel analyser tool. The signal intensity of phospho-Mypt1 was standardized to the signal intensity of Mypt1.

### Phospho-c-Jun measurement of primary neural tube cell culture

Mouse E9.5 embryos were partially digested in warm PBS containing 5 mg/ml pancreatin (SIGMA) for 3 min, followed by isolation of caudal neural tubes using fine forceps in ice-cold PBS. The isolated neural tubes was passed through a 27-gauge needle (TERUMO, Tokyo, Japan) ten times to dissociate cells, which were subsequently cultured in a 1:1 mixture of Dulbecco’s modified Eagle’s medium (GIBCO) and F-12 nutrient (GIBCO) containing 0.6% glucose, 5 mM HEPES buffer, 25 μg/ml insulin, 100 μg/ml transferrin, 20 nM progesterone, 60 μM putrescine, 30 nM selenium chloride, 10 ng/ml FGF-2, and 20 ng/ml EGF, as described by Tropepe et al.^45^. Dissociated cells were seeded onto a collagen type I (Nippi, Tokyo, Japan) coated 96-well cell culture plate (3596, Corning, NY) and cultured for 24 h at 37 °C in a humidified atmosphere containing 5% CO_2_ and 3% O_2_. Cells were subsequently fixed with 4% PFA and permeabilised with 0.1% TritonX-100, followed by blocking with PBS containing 10% FBS for 1 h at room temperature. Cells were then incubated overnight with the primary antibody at 4 °C, excess of which was removed by three washes with PBS, prior to treatment with Alexa labelled secondary antibodies. Subsequent to three washes with PBS, immunostained cells were visualized using the high-throughput high-content imaging system Opera Phenix (Perkin Elmer, Waltham, MA), and data were analysed using Harmony 4.5 (Perkin Elmer). On average 342 ± 99 cells were selected as the Nestin-positive cells in each well and mean fluorescent intensity of Alexa in the selected cells was calculated.

### Inner ear hair cell orientation analysis

The cochlea of the inner ear was isolated from mouse E18.5 embryos and attached to the transparent PET membrane of the cell culture insert (353096, Corning). They were then fixed with PBS containing 4% PFA for 1 h at room temperature, followed by three washes with PBS. Immunostained samples were visualized using a confocal microscope (LSM710; Zeiss, Oberkochen, Germany). The individual stereocilia angle was determined as previously described^35^, and measured using the Fiji angle tool. Data for different genotypes was obtained from at least 100 cells in each hair cell row of mice from three different litters.

### Statistical analysis

All data analyses were performed using Prism 9 software (GraphPad Software, San Diego, CA, USA), and mean ± standard deviation was plotted in each graph. *P*-values below 0.05 were considered statistically significant.

## Supplementary Information

Supplementary Information.

## Data Availability

The datasets generated during and/or analysed during the current study are available from the corresponding authors on reasonable request.
