# Supplementary material for: Functional interaction between Vangl2 and N-cadherin regulates planar cell polarization of the developing neural tube and cochlear sensory epithelium
Source: Sci Rep. 2023 Mar 8;13:3905. doi: 10.1038/s41598-023-30213-x (PMC9995352; doi:10.1038/s41598-023-30213-x)
Supplement: Supplementary file 1 — Supplementary Information. [file 41598_2023_30213_MOESM1_ESM.pdf]

Supplementary Information

Functional interaction between Vangl2 and N-cadherin regulates planar cell polarization of the developing neural tube and cochlear sensory epithelium

Affiliations

Tadahiro Nagaoka[1]*, Tatsuya Katsuno[2], Kyoka Fujimura[1], Kunihiro Tsuchida[1]*, Masashi Kishi[3]*

1 Division for Therapies against Intractable Diseases, Center for Medical Science, Fujita Health University, Toyoake 470-1192, Japan

2 Center for Anatomical, Pathological and Forensic Medical Researches, Graduate School of Medicine, Kyoto University, Kyoto, Kyoto 606-8507, Japan

3 Neuroscience Laboratory, Research Institute, Nozaki Tokushukai Hospital, Daito, Osaka 574-0074, Japan

*** Corresponding authors**

Email: nagaokat@fujita-hu.ac.jp (TN)

Email: tsuchida@fujita-hu.ac.jp (KT)

Email: masashi.kishi@gmail.com (MK)

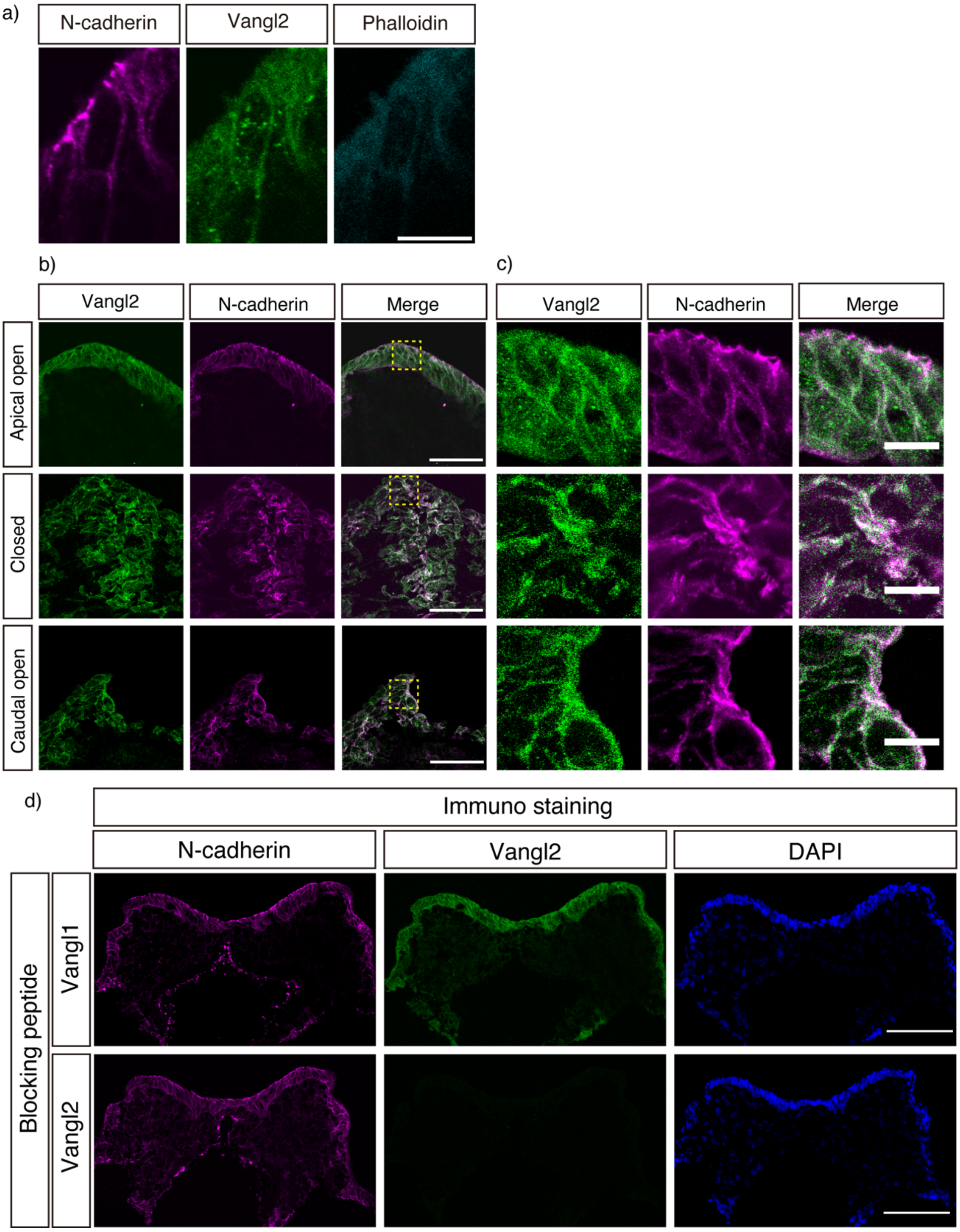

Supplementary Figure 1. Co-localization of Vangl2 and N-cadherin in neural tubes and specific immunofluorescent staining of neural tube sections by anti-Vangl2 antibody

a) Raw images of Figure 2b. b) Immunostaining of fresh frozen sections of E8.5 ICR mouse embryo with anti-Vangl2 and anti-N-cadherin antibodies confirmed co-expression and partial co-localization of the proteins. c) Magnified images of the yellow broken box in panel (b). d) The specificity of anti-Vangl2 antibodies was evaluated by competitive binding with blocking peptides (sc-515862 P; Santa Cruz Biotechnology). Anti-Vangl2 blocking peptides affected immunofluorescent intensity, whereas anti-Vangl1 blocking peptides (sc-166844 P) did not. Confocal images were taken using a 20× objective lens. Scale bars: 10 μm in a, 50 μm in b, 10 μm in c and 100 μm in d

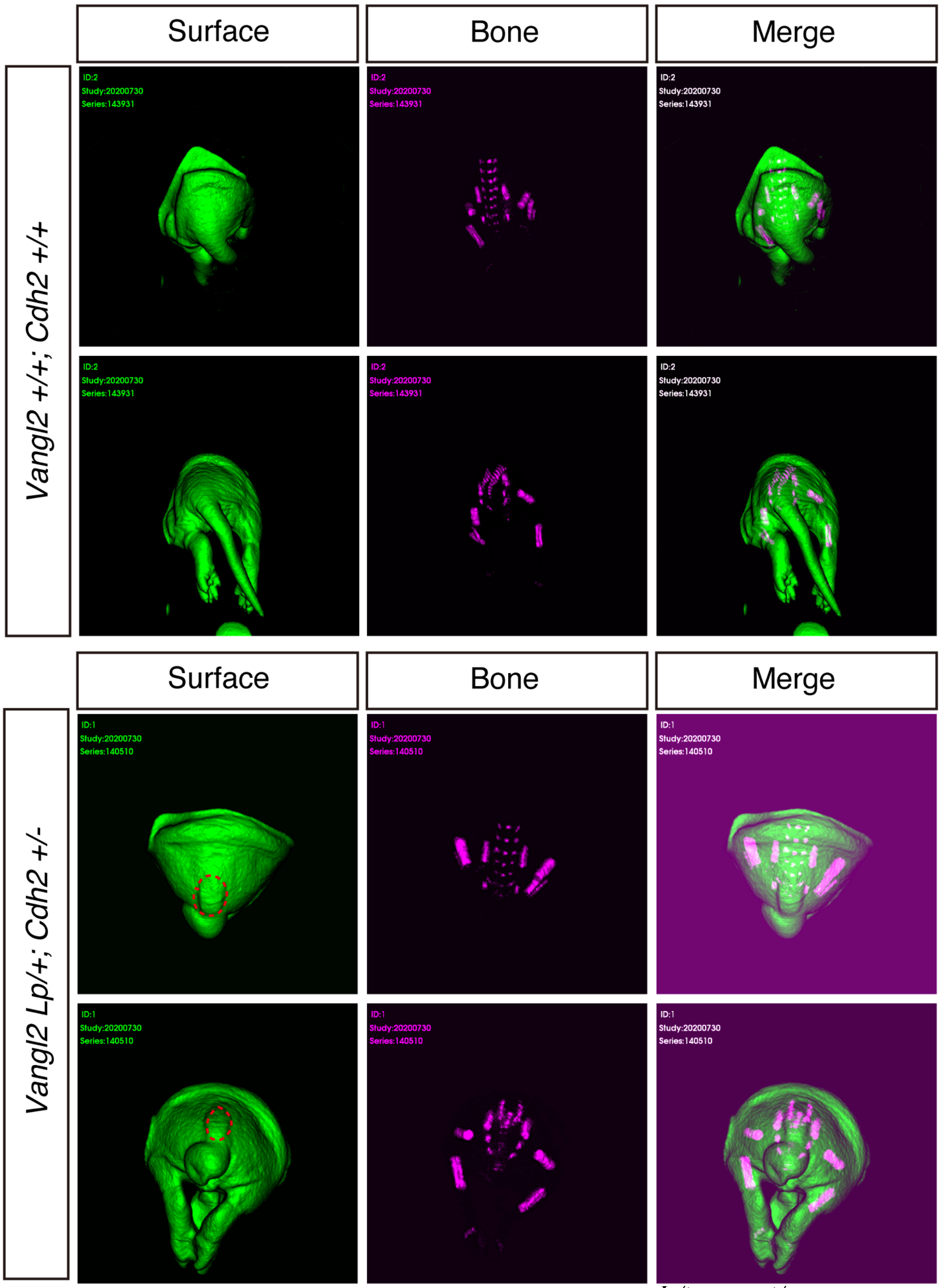

Supplementary Figure 2. Area of neural tube defect in *Vangl2$^{Lp/+}$; Cdh2$^{+/-}$* mouse embryo at E18.5

Lower bodies of *Vangl2$^{Lp/+}$; Cdh2$^{+/-}$* E18.5 mouse embryos were scanned by micro computed tomography (micro-CT). Upper panels show the dorsal view and lower panels show the caudal side view. The open spinal cord that is located around the sacrum is circled by a red broken line.

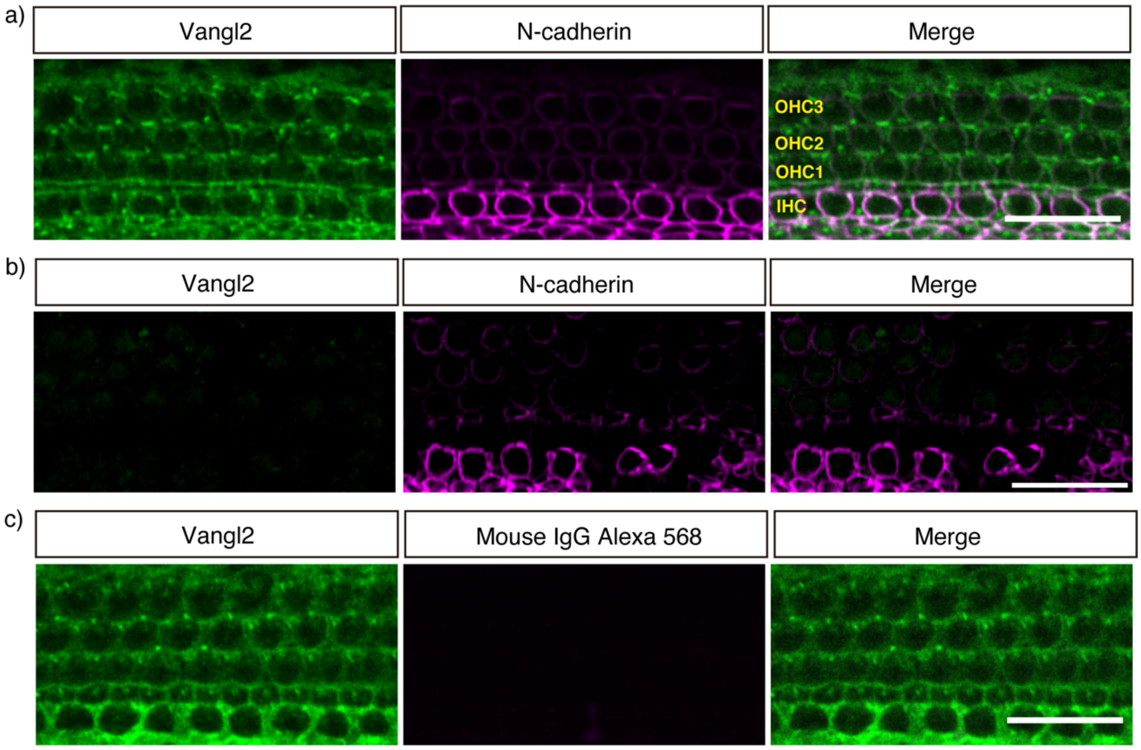

Supplementary Figure 3. Expression of Vangl2 and N-cadherin in inner ear hair cells of E18.5 mouse embryos

Isolated inner ear cochlea of E18.5 mouse embryos were fixed with ice-cold methanol before immunofluorescence analysis. a) Immunostaining analysis revealed Vangl2 expression in the outer and inner hair cells, and stronger N-cadherin expression in the inner ear hair cells than that in the outer hair cells. b) Addition of 2 μg/ml Vangl2 blocking peptide abolished the Vangl2 signal, thus indicating specificity of the anti-Vangl2 antibodies. c) Immunostaining of the cochlea with anti-Vangl2 antibodies. Confocal images were taken using a 40 X oil immersion objective lens. Scale bars: 20 μm

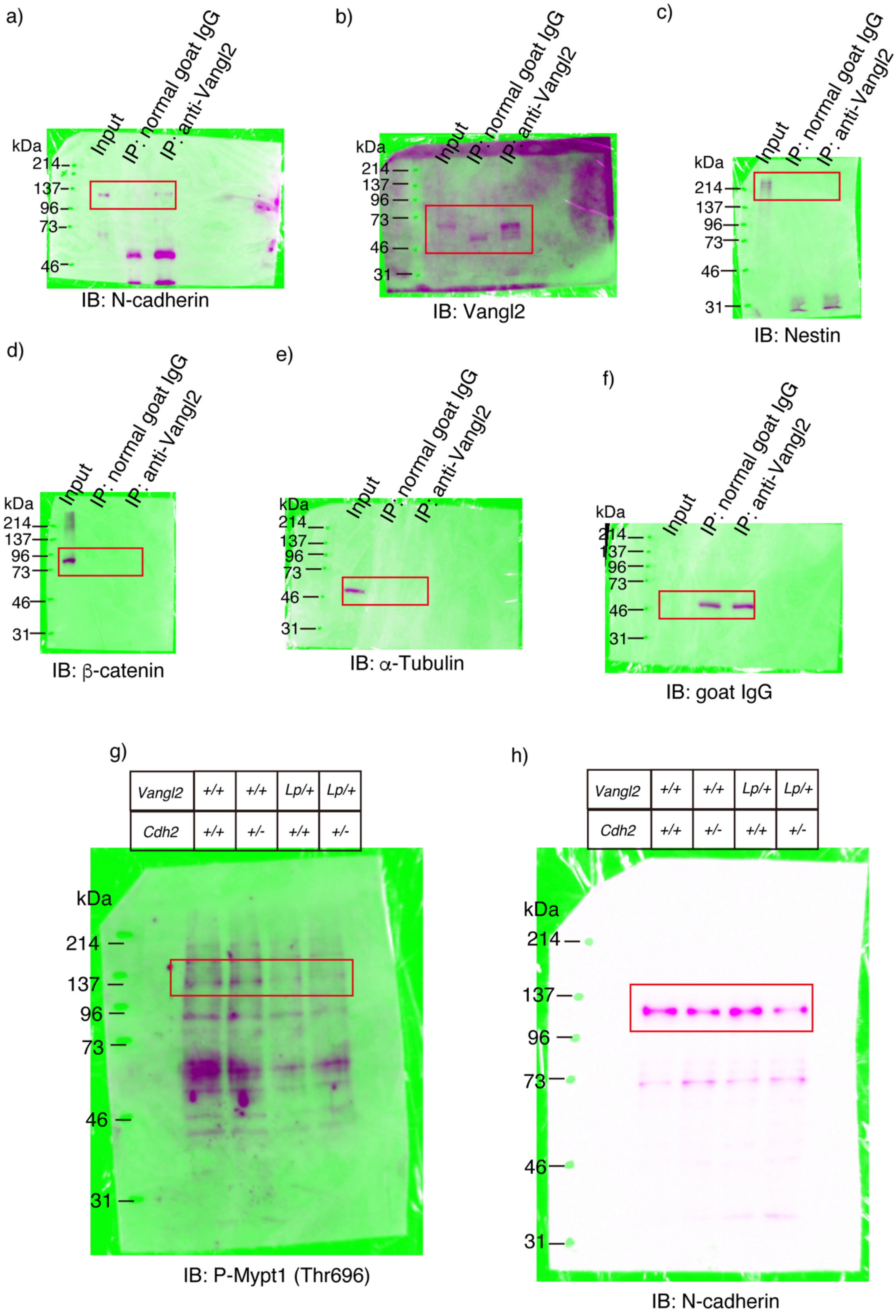
a)
Input
IP: normal goat IgG
IP: anti-Vangl2
kDa
214
137
96
73
46
IB: N-cadherin
b)
Input
IP: normal goat IgG
IP: anti-Vangl2
kDa
214
137
96
73
46
31
IB: Vangl2
c)
Input
IP: normal goat IgG
IP: anti-Vangl2
kDa
214
137
96
73
46
31
IB: Nestin
d)
Input
IP: normal goat IgG
IP: anti-Vangl2
kDa
214
137
96
73
46
31
IB: β-catenin
e)
Input
IP: normal goat IgG
IP: anti-Vangl2
kDa
214
137
96
73
46
31
IB: α-Tubulin
f)
Input
IP: normal goat IgG
IP: anti-Vangl2
kDa
214
137
96
73
46
31
IB: goat IgG
g)
Vangl2 +/+ +/+ Lp/+ Lp/+
Cdh2 +/+ +/- +/+ +/-
kDa
214
137
96
73
46
31
IB: P-Mypt1 (Thr696)
h)
Vangl2 +/+ +/+ Lp/+ Lp/+
Cdh2 +/+ +/- +/+ +/-
kDa
214
137
96
73
46
31
IB: N-cadherin

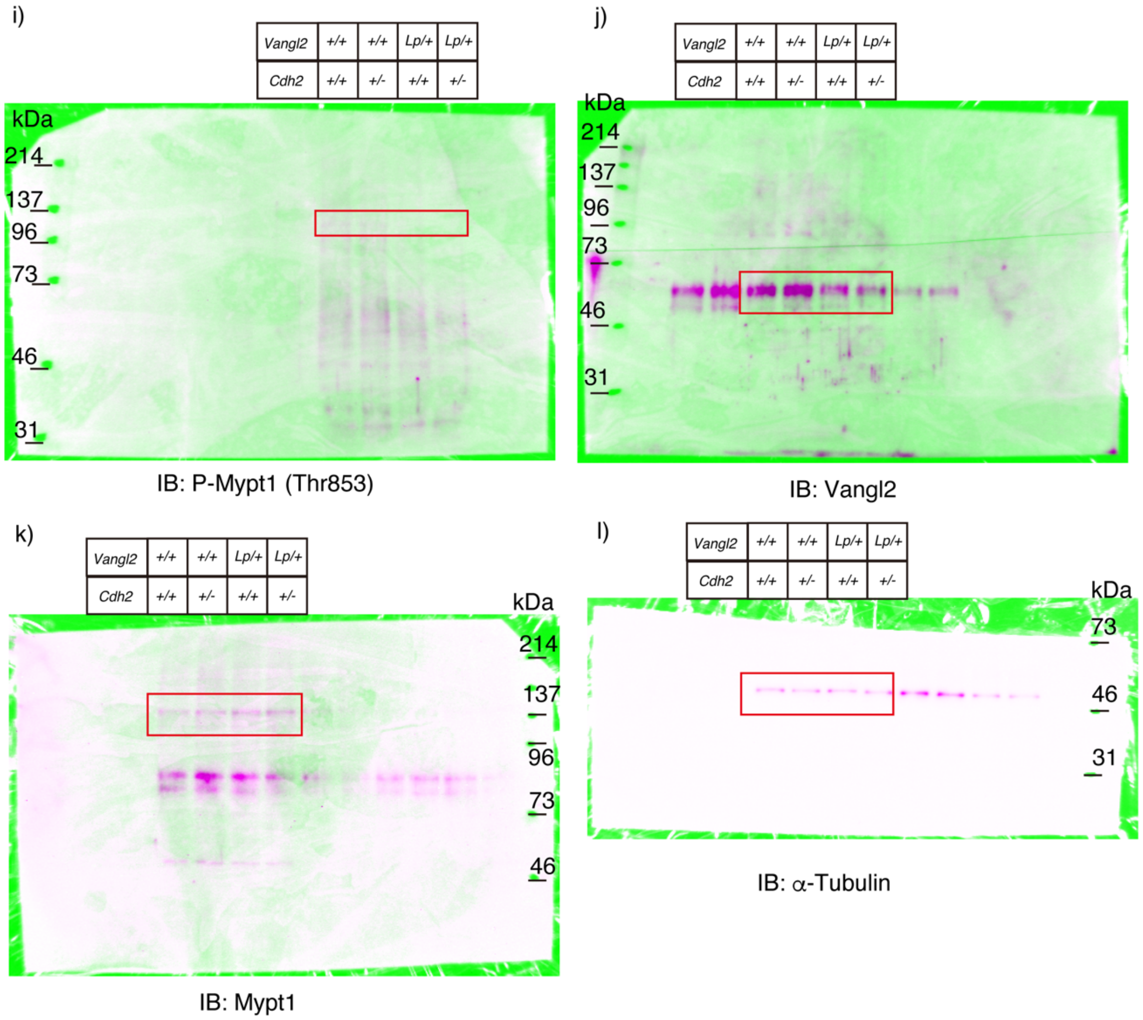

Supplementary Figure 4. Full-length images of western blot analyses
a)-f) Full length of images presented in Figure 1. g)-l) Full length of images presented in Figure 4. Red square depicts the area presented in the main figures.

Supplementary Materials and Methods

Micro-CT

Mouse E18.5 embryos were sacrificed by decapitation and cleaved at the abdominal level. The lower bodies were fixed with PBS containing 4% PFA and set on a polystyrene bed for scanning by micro-CT (R_mCT2, Rigaku, Tokyo, Japan) under the following conditions: 90 kV, 160 µA, 3 min scan time, and 30 mm FOV. Data were processed using 3DViewer built in R_mCT2, and the bony parts were displayed using the part selection function.

Supplementary Table 1. List of antibodies

Antigen	Host	Manufacturer	Clone ID or Product number	Application (Dilution)	Conjugation
Activin RIIB	Mouse	R&D systems	MAB3393	IF (1:200)	
β-catenin	Mouse	BD Biosciences	14/Beta-Catenin	WB (1:1000)	
Digoxigenin	Sheep (Fab fragment)	Roche	11093274910	in situ (1:2000)	AP
c-Jun	Rabbit	Cell Signaling Technology	60A8	IF (1:200)	
Phospho-c-Jun (Phospho-Ser63)	Rabbit	Cell Signaling Technology	E617P	IF (1:200)	
Myo7A	Rabbit	Proteus Biosciences	25-6790	IF (1:500)	
Mypt1	Rabbit	Cell Signaling Technology	D6C1	WB (1:500)	
Phsopho-Mypt1 (Phospho-Thr696)	Rabbit	Cell Signaling Technology	#5163	WB (1:500)	
Phospho-Mypt1 (Phospho-Thr853)	Rabbit	Cell Signaling Technology	#4563	WB(1:1000)	
N-cadherin	Mouse	BD Biosciences	Clone 32/N-Cadherin	WB, IF (1:1000)	
Nestin	Mouse	Thermo scientific	Rat401 (4D4)	IF (1:200)<br>WB (1:500)	
α-Tubulin	Mouse	Proteintech	66031-1-Ig	WB (1:1000)	
Vangl2	Goat	Santa Cruz	N-13	IP, IF (1:100)<br>WB (1:500)	
N.A.	Goat	Santa Cruz	sc-3887 (normal Goat IgG)	IP (1:200)	
Goat IgG	Donkey	Life Technologies	A-32814	IF (1:500)	Alexa 488
Goat IgG	Donkey	Santa Cruz	sc-2020	WB (1:5000)	HRP
Goat IgG Light Chain Specific	Mouse	Jackson ImmunoResearch	205-032-176	WB (1:5000)	HRP
Mouse IgG	Donkey	Life Technologies	A-10037	IF (1:500)	Alexa 568
Mouse IgG	Goat	Life Technologies	A-11004	IF (1:500)	Alexa 568
Mouse IgG	Goat	Bio-Rad	170-6516	WB (1:5000)	HRP
Rabbit IgG	Goat	Life Technologies	A-11008	IF (1:500)	Alexa 488
Rabbit IgG	Goat	Santa Cruz	sc-2030	WB (1:5000)	HRP
